# Malseated Liner in Modular Dual Mobility Total Hip Replacement: A Report of Three Cases

**DOI:** 10.7759/cureus.60437

**Published:** 2024-05-16

**Authors:** Caleb D Gerber, Anderson Lee, Vihan De Silva, David Yatsonsky, Gregory M Georgiadis

**Affiliations:** 1 Department of Medicine, The University of Toledo College of Medicine and Life Sciences, Toledo, USA; 2 Department of Orthopedic Surgery, The University of Toledo Medical Center, Toledo, USA; 3 Department of Orthopedic Surgery, ProMedica, Toledo, USA

**Keywords:** osteoarthritis (oa), total hip arthroplasty (tha), dual mobility total hip arthroplasty, malseated liner, dual mobility

## Abstract

Modular dual mobility total hip arthroplasty (THA) can be associated with complications if the liner is malseated, which can be unappreciated intraoperatively. A meticulous surgical technique is needed to ensure that the liner is perfectly seated. In addition, a malseated liner can be missed if the postoperative films are not carefully reviewed by the surgeon.

We present three cases of THA associated with a malseated modular dual mobility liner. In one case, the malpositioned liner was appreciated intraoperatively, but it was wedged in place and could not be removed. The entire shell needed to be revised. In two other cases, malseating was not detected intra-operatively. Both were appreciated postoperatively, and early revision surgery was needed.

## Introduction

Dual mobility (DM) articulations in total hip arthroplasty (THA) were first described by Bousquet in the 1970s [1]. Their use in THA is steadily increasing [2]. The primary indication has been to decrease the incidence of dislocation [3]. DM is often used in revision surgery [4], but indications are constantly expanding. These may include any condition that may predispose a patient to dislocation such as stiff spine or back fusion, excessive soft tissue laxity, neurologic disease, femoral neck fracture, and hip dysplasia. Some surgeons have begun the use of DM in routine primary THA. Not all these constructs are the same. Modular DM can result in malseating and may be a potential source of implant failure. Malseating has been associated with an increased chance of impingement and accelerated corrosion and is a risk factor for complete liner dissociation [2].

Between 2018 and 2019, a consecutive review of the senior author’s first 30 DM THA using the Zimmer Biomet (Warsaw, IN) G7 acetabular system revealed three patients (10%) with malseated liners. The cases are described below.

## Case presentation

Case histories

Case 1

In 2019, a 56-year-old osteoporotic woman developed post-traumatic arthritis of her left hip after open reduction and internal fixation of a low-energy acetabular fracture that was fixed through an anterior ilioinguinal approach. She also had previous lower lumbar spine fusion. She had a left THA through a posterior approach, with the placement of a 52 mm diameter multi-hole Zimmer Biomet G7 cup with multiple screws, a 42 mm cobalt chrome modular liner, and a 42 mm E poly/28 mm ceramic DM articulation, and a Biomet (Warsaw, IN) size 14 Taperloc stem.

**Figure 1:**
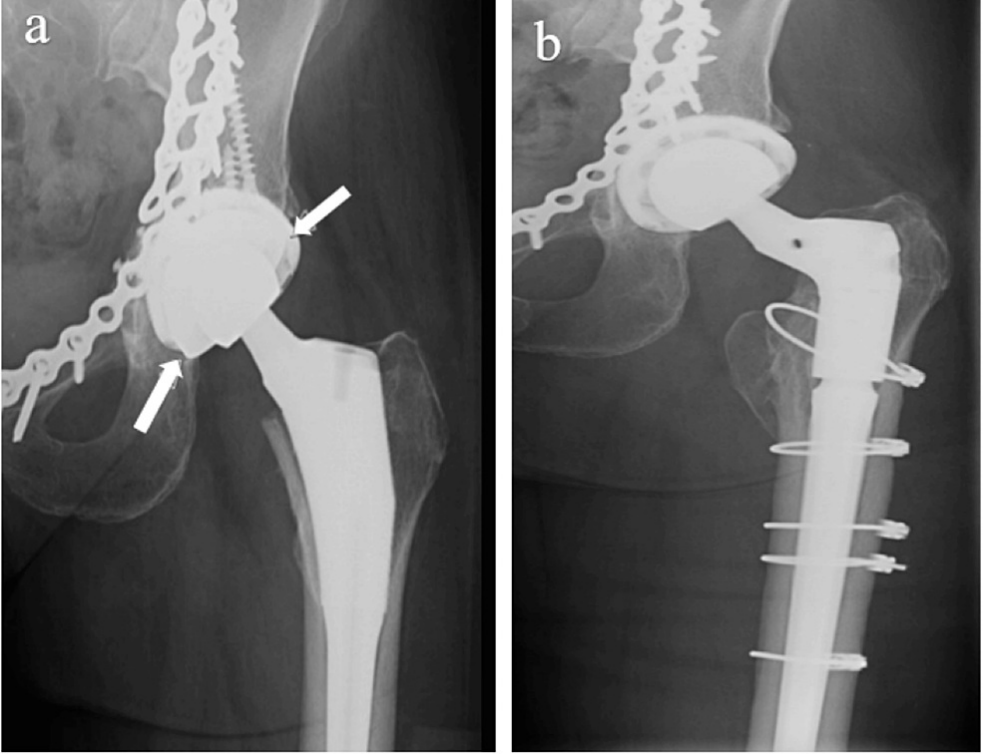
Pre- and postoperative anteroposterior radiograph after revision of malseated DM liner. (A) Malseated DM liner of a Biomet G7 cup on an anteroposterior hip view, with arrows illustrating the malpositioned CoCr articulation. (B) Post complete revision with modular fluted stem and 36 mm ceramic head/UHMWPE liner. DM: dual mobility; CoCr: cobalt chrome; UHMWPE: ultra-high molecular weight polyethylene.

Postoperatively, she was allowed to weight bear as tolerated. Her malseated cobalt chrome liner was not appreciated during her initial hospitalization but did become apparent in the early postoperative period (Figure 1A). Revision surgery was recommended, but initially, the patient appeared to be doing well and decided to defer any subsequent surgery. However, she began experiencing pain and was revised at three months.

At the time of her revision, extensive metallosis was found. This deposition and accumulation of metallic particles originating from the cobalt chrome liner and the titanium alloy outer shell results in the development of adverse local tissue reaction (ALTR), and in some cases previously has been associated with systemic effects. To gain better access to the cup, the Taperloc stem was removed. Her cobalt chrome liner was grossly loose and was removed. All the screws in her multi-hole cup were removed. The acetabular shell appeared stable, and a 36 mm internal diameter vitamin E-impregnated ultra-high molecular weight polyethylene (UHMWPE) liner was inserted in the cup with an accompanying 36 mm ceramic head. This was the attending surgeon's decision to avoid any further possible metal ion release (as opposed to inserting another modular DM liner).

A new Taperloc stem was implanted. Postoperatively, she was found to have a peri-prosthetic fracture around her stem. This was felt to have been an unappreciated intra-operative complication of the removal of a stem that had early ingrowth. The patient was taken back to the operating theater two days later where a Biomet Arcos modular revision stem was placed (Figure 1B). The patient was allowed to weight bear as tolerated and recovered well from that point. At a 45-month follow-up, she was walking without a limp, had no pain, and was pleased with her outcome.

Case 2

**Figure 2:**
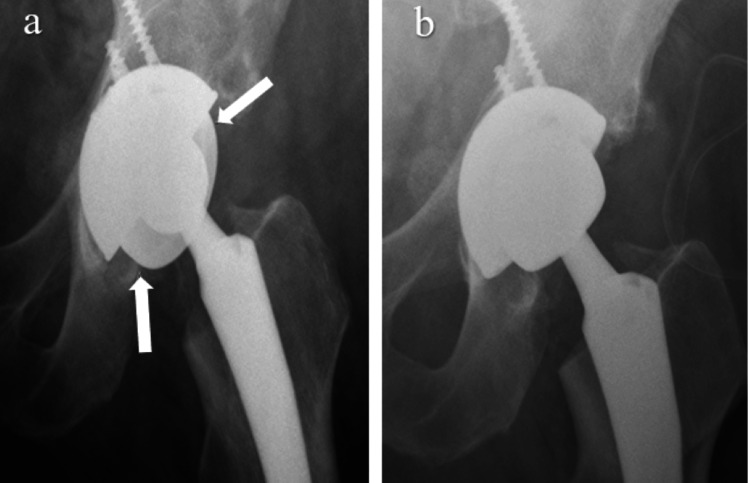
Pre and postoperative films of grossly malpositioned DM liner and revision. (A) Gross disengagement of the DM modular CoCr liner in a Biomet G7 cup, with arrows illustrating the outline of the loose liner. (B) Post revision of the DM liner with a 40 mm ceramic head/UHMWPE liner. DM: dual mobility; CoCr: cobalt chrome; UHMWPE: ultra-high molecular weight polyethylene.

In 2019, a 74-year-old man with multiple medical comorbidities (chronic kidney disease, chronic obstructive pulmonary disease, coronary artery disease, morbid obesity) underwent a left total hip replacement utilizing a posterior approach. A Zimmer Biomet G7 60 mm diameter four-hole cup with selected screws was inserted, with a 46 mm cobalt chrome DM modular liner and a 46 mm vitamin E UHMWPE/28 mm ceramic DM articulation. A Biomet size 16 Taperloc stem was used. The immediate postoperative films showed an obvious disassociation of the DM liner (Figure 2).

The patient was returned to the operating theater where that DM articulation was removed and a 40 mm internal diameter vitamin E-impregnated UHMWPE liner was inserted in the cup with an accompanying 40 mm ceramic head. The patient was allowed to weight bear as tolerated. At 47-month follow-up, the patient was having significant medical issues (heart failure, cardiomyopathy, chronic kidney disease), but his left hip was performing well. The patient's comorbidities did not affect the implant selection.

Case 3

**Figure 3:**
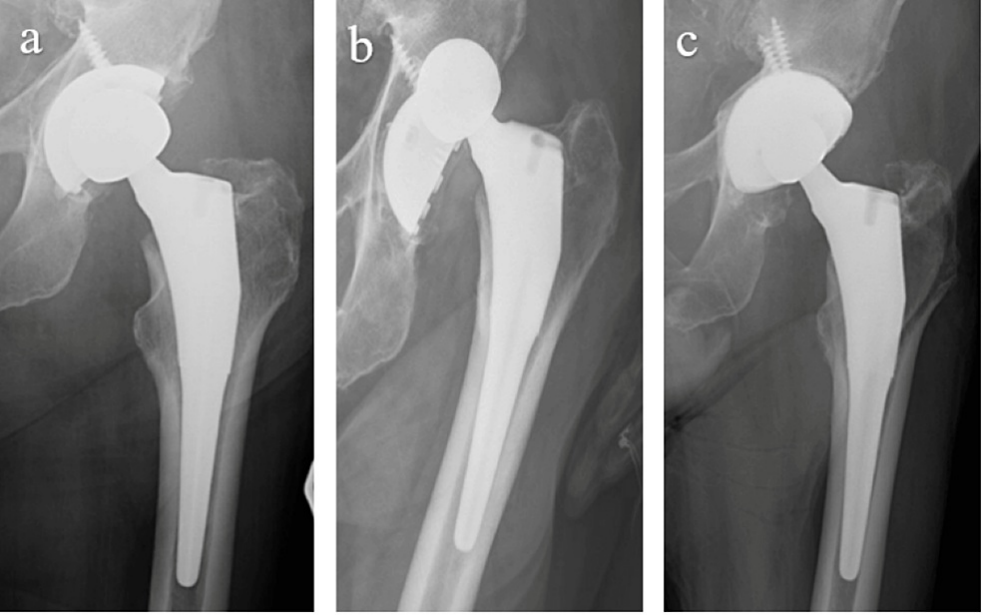
Chronic total hip dislocation treated with DM. (A) Initial cementless Biomet THA with acetabular malpositioning. (B) Documented prosthetic dislocation. (C) Revision with a larger, more optimal cup and DM construct. DM: dual mobility; THA: total hip arthroplasty.

In 2016, an 80-year-old man underwent a left total hip replacement for osteoarthritis (Figure 3A). A Biomet 56 mm diameter Mallory-Head cup, with a 36 mm UHMWPE liner, a 36 mm cobalt chrome metal head, and a size 15 Taperloc stem were implanted.

He initially did well, but over the course of the next three years, he experienced three prosthetic hip dislocations (Figure 3B), each one being treated with a closed reduction. In 2019, he was taken to the operating room for revision of his acetabular cup, which was felt to be malpositioned and was supposed to be the underlying cause of his recurrent dislocations.

The old acetabular shell was removed. A larger, more anteverted Biomet G7 multi-hole cup was inserted. A larger, more optimally positioned cup with a DM liner was felt to be the best solution for this patient’s instability. The cobalt chrome dual mobility liner was inserted into the titanium shell. After impaction, it was noted that the liner was not seated properly with a significant overhang. The liner could not be disengaged, and the entire shell was removed. A new 58 mm diameter G7 multi-hole cup with multiple screws was inserted and a new DM liner was placed, with a 46 mm vitamin E UHMWPE/28 mm ceramic articulation (Figure 3C). The patient was allowed to weight bear as tolerated, without any restrictions. His DM prosthetic hip was performing well at the 54-month follow-up.

## Discussion

DM in THA consists of two articulations. The first involves a UHMWPE liner that articulates with the acetabular component, and a smaller femoral head that engages inside the liner. This results in a larger head side and two separate articulations. The increased head-to-neck ratio increases the translation distance required for femoral head dislocation, resulting in a more stable construct [5].

Not all DM constructs are equivalent. At one point, it was estimated that there were almost 40 different DM cups in the French market where this concept was first developed [6]. In the United States, the Zimmer Biomet G7 system (Warsaw, IN) and the Stryker Modular Dual Mobility System (Mahwah, NJ) are popular modular systems where a cobalt chrome (CoCr) inner liner articulates with a titanium alloy shell. Smith & Nephew (Memphis, TN) has incorporated an Oxinium liner instead of CoCr into their product line. Other systems like the Stryker Anatomic Dual Mobility (Mahwah, NJ) or the Medacta (Castel San Pietro, Switzerland) are nonmodular systems.

The Zimmer Biomet modular DM system has a plastic disposable guided ring around the CoCr liner that is supposed to help guide the construct symmetrically into the titanium alloy shell. As can be seen in the study, the process can be prone to failure if meticulous technique is not performed. A malseated liner leads to early metallosis (Case 1), gross instability (Case 2), or cold welding preventing the disimpaction of the modular liner (Case 3).

Introducing DM into a THA can be associated with many potential adverse effects. These include fretting, corrosion, metallosis, elevated ion levels, intraprosthetic dislocation, and the need for revision surgery [7-12]. Intuitively malpositioning of the modular shell of a DM THA would be expected to increase these complications. The incidence of malseating modular total hip components has been reported to be anywhere from 1.3% to 16.4% [13-15]. Several authors have reported that malseating of a modular DM liner has not been a clinical problem [16-18]. However, there is still cause for concern due to the non-uniform types of DM implants, relatively small numbers, and lack of long-term follow-up. This emphasizes the importance of larger patient cohorts and registries.

The primary reason for the malseating of a modular DM construct is the technical insertion of the articulating metal liner. Large body habitus, difficult exposure that does not allow full visualization of the cup, soft tissue interposition, prominent screws in the cup, asymmetric impaction, deformation of the shell, and surgeon inexperience with the technique are all potential causes. Special attention should be taken to ensure proper modular liner insertion so that malseating does not occur. Wide exposure to allow for circumferential exposure of the cup and following manufacturer recommendations are critical, as well as adherence to stable combined component version. In our small series, one malseated liner was appreciated in surgery and could not be removed after impaction. A decision was made to revise the entire cup at that time.

This represents an initial experience with a specific DM system. As a result of this initial experience, the senior author has decreased his use of DM and increased his use of larger diameter (40 mm) ceramic heads when clinically indicated [19]. However, DM remains a useful technique that needs to be in the armamentarium of every revision hip surgeon, as there may not be another satisfactory solution for certain difficult cases. Future advances will likely occur in newer implant designs and material sciences.

## Conclusions

Malseated a modular liner in DM THA remains a problem and may be an issue with surgeons inexperienced in the technique. Special attention should be given to the surgical exposure and manufacturer recommendations, making sure the metal liner is well seated. Postoperative radiographs should also be carefully evaluated as the surgeon may choose to more closely follow a patient with a malseated modular DM cup. If the surgeon has any concerns about malseating of a DM THA, additional radiographs (Judet views) or advanced imaging (metal reducing CT) should be considered.
